# Gut microbiota-mediated uric acid metabolism: Novel insights for developing urate-lowering and anti-gout medications

**DOI:** 10.1016/j.pscia.2026.100148

**Published:** 2026-09-01

**Authors:** Yuxin Jiang, Xiaoyu Shi, Qian Yang, Jianxin Pang, Peng Zhan, Shujing Xu, Ting Wu

**Affiliations:** a Department of Medicinal Chemistry, State Key Laboratory of Discovery and Utilization of Functional Components in Traditional Chinese Medicine, Shandong Key Laboratory of Druggability Optimization and Evaluation for Lead Compounds, School of Pharmaceutical Sciences, Shandong University, 44 West Culture Road, Jinan, Shandong, 250012, China; b NMPA Key Laboratory for Research and Evaluation of Drug Metabolism &Guangdong Provincial Key Laboratory of New Drug Screening & Guangdong-Hongkong-Macao Joint Laboratory for New Drug Screening, School of Pharmaceutical Sciences, Southern Medical University, Guangzhou, 510515, China; c State Key Laboratory of Natural and Biomimetic Drugs, Peking University, Xue Yuan Rd. 38, Beijing, 100191, China; d State Key Laboratory of Druggability Evaluation and Systematic Translational Medicine (Tianjin Institute of Pharmaceutical Research), Tianjin, China; e School of Health and Life Sciences, University of Health and Rehabilitation Sciences, Qingdao, 266001, China

**Keywords:** Gut microbiota, Uric acid metabolism, Hyperuricemia, Gout, Urate-lowering drugs

## Abstract

Hyperuricemia, caused by purine metabolism dysfunction and impaired uric acid excretion, is increasing in prevalence globally. Current urate-lowering drugs have limitations in safety and applicability. The gut, which is central to uric acid production and excretion, hosts microbiota closely linked to uric acid metabolism and gout-related inflammation. This paper summarizes advances from PubMed and CNKI (2012–March 2026) on microbiota-mediated regulation of uric acid metabolism and gout. Gut microbiota promote hyperuricemia and gout *via* multiple mechanisms: regulating purine metabolism and uric acid production, degrading urate, modulating intestinal urate transport, and activating inflammatory pathways. We further classify microbiota-targeted urate-lowering interventions into four categories: (i) microbial metabolites and functional strains, (ii) microbiota-modulating natural products, (iii) microbiota-utilizing polysaccharides, and (iv) bioactive peptides. These strategies have unique advantages including high intestinal exposure, microbiota responsiveness, structural diversity, and multi-pathway regulation potential, acting *via* key axes such as microbiota–PPARγ–ABCG2 and TLR4/NF-κB–NLRP3. By targeting systemic regulation instead of simple transporter inhibition, they fill critical gaps in current therapy. From a medicinal chemistry perspective, these molecules are promising prototype templates for safer, more effective anti-hyperuricemic and anti-gout drugs.

## List of abbreviations

HUA—: Hyperuricemia
UA—: uric acid
XOD—: xanthine oxidase
GA—: gouty arthritis
GLUT9—: glucose transporter 9
ABCG2—: ATP-binding cassette transporter G2
PPARγ—: peroxisome proliferator-activated receptor γ
PDB—: purine-degrading bacteria
PI3K/AKT/mTOR—: phosphatidylinositol 3-kinase/Protein Kinase B/mammalian target of rapamycin
DOPDH—: dioxopurine dehydrogenase
LPS—: lipopolysaccharide
SCFAs—: short-chain fatty acids
DOPOR—: dioxopurine oxidoreductase
IL-10—: interleukin-10
TLR4—: Toll-like receptor 4
IL-1β—: interleukin-1β
NLRP3—: NOD-like receptor family pyrin domain-containing 3
BBR—: Berberine
ZPG—: Zhejiang Plantaginis Semen glycosides
VCP—: viscum coloratum polysaccharide
CSF—: *Capparis spinosa* fruit
FC—: *Fagonia cretica*
TMAO—: trimethylamine *N*-oxide
Cr—: creatinine

## 1 Introduction

Hyperuricemia (HUA) is a common metabolic disorder caused by an imbalance in the production and/or excretion of uric acid (UA) [1]. In recent years, its global prevalence has risen rapidly, making it one of the most common metabolic disorders after diabetes and obesity [2]. Beyond gout, HUA is also closely associated with multiple conditions, including chronic kidney disease, cardiovascular disease, and metabolic syndrome, posing a serious threat to global public health [[3], [4], [5]]. Although small-molecule urate-lowering drugs such as xanthine oxidase (XOD) inhibitors (e.g., Allopurinol, Febuxostat) and urate transporter modulators (e.g., Benzbromarone, Lesinurad) are available for clinical use, there are still limitations regarding their long-term safety, tolerability, and suitability, particularly in patients with renal impairment [6]. There is an urgent need to develop safer and more effective therapeutic strategies.

In recent years, the regulatory role of the gut microbiota in metabolic diseases has increasingly become a focus of research in this field. The gut serves not only as a vital organ for nutrient absorption but also as a significant site for the production and excretion of UA in the human body. Research indicates that approximately one-third of UA is excreted through the gut, and gut microbiota participate in maintaining UA homeostasis through multiple pathways, including regulating purine metabolism, influencing UA transporter expression, and modulating immune-inflammatory responses [24,25].

Although previous studies have demonstrated that various natural small molecules and microbiota modulators can regulate UA metabolism by influencing the gut microbiome, systematic summaries and analyses of the structure-activity relationships, mechanisms of action, and drug-likeness of these molecules remain lacking. To address the research gaps in the systematic mechanistic dissection and precise classification of active molecules for gut microbiota–targeted uric acid-lowering interventions, this mini-review systematically examines the mechanisms and molecular characteristics by which gut microbiota-derived small-molecule compounds and natural products regulate UA homeostasis. It further explores novel directions for uric acid-lowering drug development based on microbiota-small molecule regulatory pathways. This review aims to provide a theoretical basis for innovative drug development targeting diseases related to UA metabolism and to promote the application of gut microbiota-mediated small molecules in the discovery of urate-lowering drugs.

## 2 How gut microbiota regulate uric acid metabolism

UA is the final oxidation product of purine metabolism in humans. During primate evolution, inactivation of the uricase gene resulted in higher serum UA levels compared with other mammals, predisposing humans to UA accumulation and hyperuricemia [26]. In recent years, studies indicate that the maintenance of UA homeostasis in the human body is determined by a dynamic balance among uric acid production, metabolism, and excretion [27]. Dietary purines are metabolized through complex pathways involving both host tissues, particularly the intestine and liver, and microbial communities. Excessive uric acid production is mainly associated with increased purine degradation and XOD-mediated oxidation, whereas urate elimination is primarily achieved through renal and intestinal excretion pathways. Notably, approximately one-third of uric acid is eliminated through the intestine, where gut microorganisms contribute to urate degradation and regulation of intestinal urate transport.

**Fig. 1:**
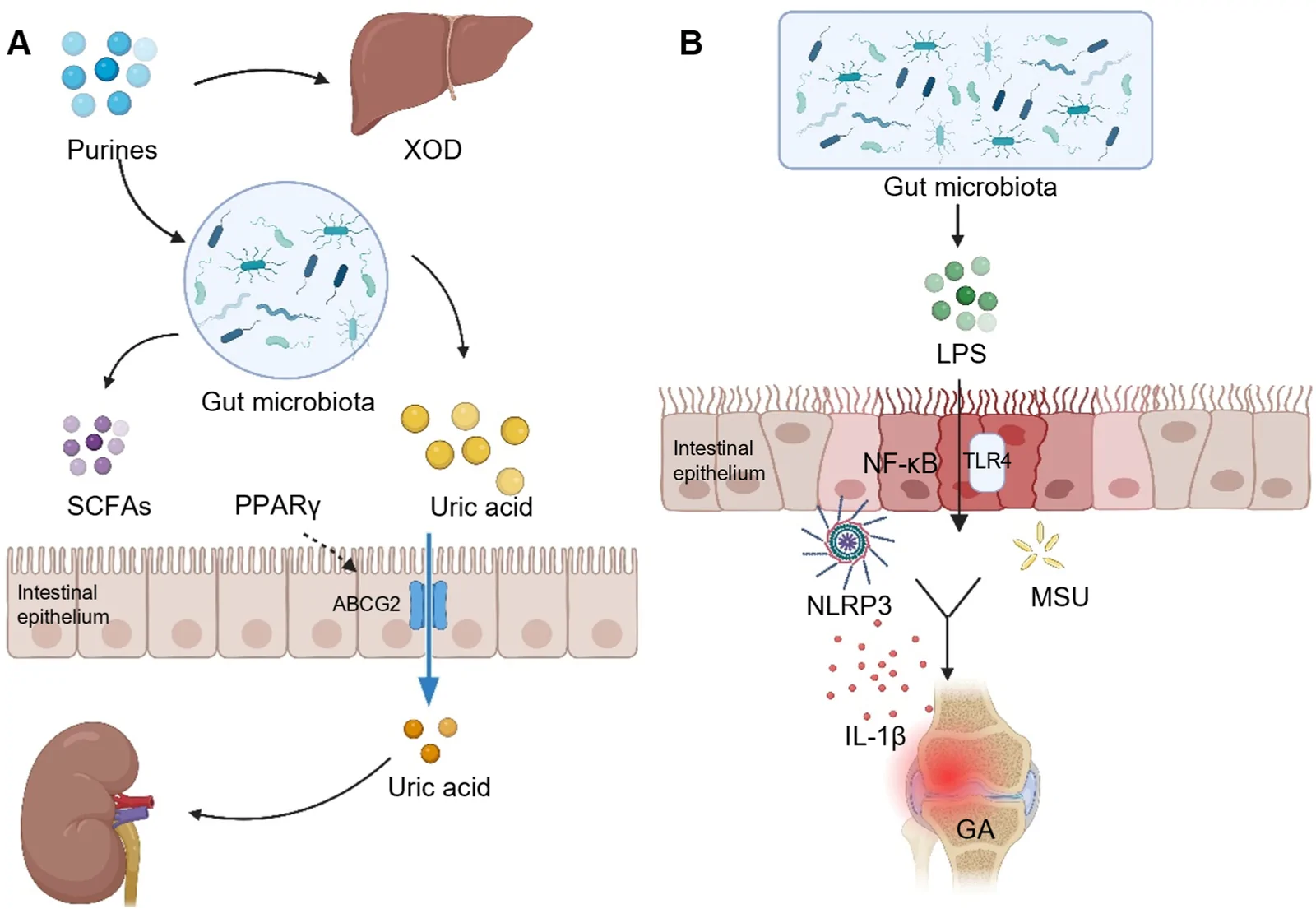
The representative mechanisms of gut microbiota-mediated regulation of UA metabolism and gouty inflammation. (A) Metabolic mechanism: Gut microbiota-derived metabolites, such as SCFAs, activate PPARγ signaling to upregulate ABCG2 expression and promote intestinal urate excretion; (B) Immune mechanism: Gut dysbiosis increases LPS translocation, activating TLR4/NF-κB and NLRP3 signaling pathways to trigger IL-1β release and gouty inflammation. This figure was created using BioRender (https://biorender.com).

Increasing evidence indicates that the gut microbiota participates in uric acid homeostasis through multiple mechanisms, including modulation of purine metabolism and XOD activity, production of microbiota-derived metabolites, regulation of intestinal urate transporters such as ABCG2 and GLUT9, and enrichment of uricolytic bacteria capable of degrading urate through microbial enzymatic pathways [28]. In addition, gut microbiota-derived signals, including lipopolysaccharide (LPS)-mediated inflammatory pathways, can influence gout progression by regulating immune responses, although these inflammatory mechanisms do not necessarily directly affect serum uric acid levels (Fig. 1).

Therefore, gut microbiota may regulate hyperuricemia and gout through two interconnected but distinct dimensions: (i) direct regulation of uric acid levels by affecting its production, degradation, and excretion; and (ii) modulation of inflammatory responses that contribute to gout initiation and progression without necessarily altering UA concentrations.

### 2.1 Gut microbiota dysbiosis leads to abnormal uric acid metabolism

The metabolism of dietary purines primarily occurs in the gut. Pancreatic nucleases break down dietary nucleic acids into mononucleotides and polynucleotides, which are then further processed by small intestinal epithelial cells and associated enzymes into absorbable purine bases [29]. The concentrative nucleoside transporter at the apical surface of intestinal epithelial cells facilitates the uptake of purine nucleosides into cells. Simultaneously, nucleotides released during the self-renewal of intestinal epithelial cells also contribute to the dietary purine load. These purines are subsequently transported to the liver, where they are converted to UA by XOD and released into the bloodstream. Additionally, XOD is expressed locally in the intestine, where it directly catalyzes the oxidation of xanthine and hypoxanthine within the intestinal microenvironment, thereby mediating the *in situ* production of uric acid in the gut.

Regarding UA excretion, intestinal epithelial cells express a variety of uric acid transporters, such as glucose transporter 9 (GLUT9) and ATP-binding cassette transporter G2 (ABCG2). Among them, ABCG2 is highly expressed in the small intestine and colon, serving as a key transporter for intestinal uric acid excretion [7]. However, the development of direct ABCG2-targeted urate-lowering drugs faces great challenges and high off-target risk due to ABCG2's complex protein structure, significant transmembrane dynamics and wide tissue distribution. At present, there are no clinically available direct ABCG2 inhibitors or activators, so research focus has gradually shifted to its upstream transcriptional regulation, to indirectly upregulate intestinal ABCG2 expression and promote uric acid excretion. Given that the transcription of ABCG2 is regulated at multiple levels, targeting its upstream transcriptional regulation has become an important entry point for improving uric acid metabolism. Among these, peroxisome proliferator-activated receptor γ (PPARγ) is a key transcription factor that can drive the expression of ABCG2 [30]. Gut microbiota-derived metabolites, including butyrate, conjugated linoleic acid, and certain secondary bile acids, have been reported to act as signaling molecules/ligands of PPARγ. Upon activation, PPARγ enhances its transcriptional activity at the promoter region of ABCG2, thereby upregulating ABCG2 expression. The increased expression of ABCG2 facilitates the active efflux of uric acid from intestinal epithelial cells into the intestinal lumen [31].

Researchers have found that approximately 15% to 20% of gut bacteria can anaerobically degrade uric acid, and these are known as purine-degrading bacteria (PDB) [27]. These gut bacteria represented by *Clostridium sporogenes* and *Escherichia coli* convert uric acid into 2,8-dioxopurine *via* selenium-dependent 2,8-dioxopurine dehydrogenase (DOPDH), and then sequentially catalyze uric acid into short-chain fatty acids (SCFAs) such as acetic acid and butyric acid through seven enzymes including 2,8-dioxopurine oxidoreductase (DOPOR). This process compensates for the physiological deficiency of uricase in humans [32]. In addition to such purine-degrading bacteria that directly decompose uric acid, some probiotics in the gut, such as *Lactobacillus*, are also involved in the regulation of uric acid production through indirect pathways. While *Lactobacillus* itself lacks the ability to directly degrade uric acid, it can indirectly reduce uric acid production by breaking down purine nucleosides. Additionally, microbial metabolites such as bile acids, trimethylamine *N*-oxide, and SCFAs can also activate the phosphatidylinositol 3-kinase/Protein Kinase B/mammalian target of rapamycin (PI3K/AKT/mTOR) and the aforementioned PPARγ signaling pathways to influence renal function and uric acid excretion capacity [33].

Patients with hyperuricemia commonly exhibit significant dysbiosis of the gut microbiota, characterized primarily by reduced abundance of *Firmicutes* (rich in PDB), an increased proportion of *Bacteroides*, and diminished capacity to produce SCFAs [34]. These alterations may lead to impaired purine degradation, increased production of XOD and lipopolysaccharide (LPS), and downregulation of UA transporter expression in the gut. Ultimately, this results in increased UA production and reduced excretion, accelerating the progression of HUA.

Impaired production of SCFAs by the gut microbiota leads to significant downregulation of GLUT9 and ABCG2 in intestinal epithelial cells. Concurrently, the activity of metabolism-related enzymes, such as hydrolases and uricase, is reduced, resulting in decreased uric acid excretion and lower excretion efficiency [35]. Notably, LPS, as a key pro-inflammatory factor, can trigger immune-inflammatory responses when abnormally elevated, thereby further contributing to the pathogenesis of GA [36,37].

### 2.2 Gut microbiota dysbiosis in hyperuricemic patients induces immune responses

Apart from producing and excreting UA, gut microbiota can also contribute to the development of hyperuricemia and gouty arthritis by regulating immune-inflammatory responses. Researchers have shown that dysbiosis can amplify inflammatory responses and promote the development of GA through three key pathways: alterations in microbial composition, disruption of metabolic products, and impairment of the intestinal barrier function [6]. These pathways collectively form a network of microbiota-metabolite-immune interactions.

Alterations in gut microbiota composition can indirectly modulate the global expression of inflammatory factors in the host by regulating the levels of microbial metabolites and intestinal barrier function. Under physiological conditions, beneficial bacteria such as *Bifidobacterium* and *Lactobacillus* maintain immune homeostasis, and they inhibit aberrant immune activation by promoting the expression of anti-inflammatory cytokines, including interleukin-10 (IL-10) and transforming growth factor-β [6,38]. In the context of hyperuricemia, the abundance of such beneficial bacteria decreases, while the proportion of Gram-negative bacteria (e.g., certain strains of *Bacteroides* and *Escherichia coli*) increases, leading to a massive release of their cell wall component LPS. LPS can activate macrophages and neutrophils *via* Toll-like receptor 4 (TLR4), thereby inducing the secretion of pro-inflammatory cytokines such as interleukin-1β (IL-1β) and ultimately triggering inflammatory responses. Furthermore, LPS acts as a priming signal for inflammasome activation, upregulating the expression of NOD-like receptor family pyrin domain-containing 3 (NLRP3) and its precursor IL-1β. When secondary signals such as urate crystals are present, they further promote NLRP3 inflammasome assembly and the maturation and release of IL-1β, thereby exacerbating the inflammatory response in GA [39].

Dysregulation of gut microbial metabolites serves as a crucial intermediate link in immune-inflammatory responses, with abnormal SCFA expression exerting the most prominent impact. As key metabolic products of the gut microbiota, SCFAs play a vital role in maintaining immune homeostasis. By activating G protein-coupled receptors (GPCRs) or inhibiting histone deacetylase activity, SCFAs can promote the differentiation of regulatory T cells and downregulate inflammatory signaling pathways, such as nuclear factor-κB (NF-κB), thereby attenuating inflammatory responses [40]. However, in patients with hyperuricemia, the reduction in SCFA-producing microbiota and decreased levels of key metabolites, such as butyrate, weaken anti-inflammatory signaling and enhance pro-inflammatory responses [41,42]. Furthermore, certain microbial metabolites such as indole derivatives and secondary bile acids can regulate the differentiation of helper T cells (Th17 cells) or influence immune cell signaling pathways, thereby further worsening the inflammatory microenvironment [43,44].

Impairment of the intestinal barrier function is a pivotal step in driving the systemic spread of inflammatory responses, with the accumulation of harmful metabolites induced by gut microbiota dysbiosis acting as the primary trigger. Under the state of microbiota dysbiosis, the abnormal accumulation of detrimental metabolites such as excessive hydrogen sulfide and the buildup of oxidative stress-related molecules directly damages the structure of intestinal epithelial cells and impairs the integrity of the intestinal mucosal barrier. Following intestinal barrier disruption, microbial-associated molecular patterns such as LPS enter the circulatory system, bind extensively to peripheral immune cells, and systemically activate inflammatory signaling pathways. This leads to a sustained release of inflammatory cytokines, further worsening urate crystal-mediated immune response activation [8].

Overall, the gut microbiota plays a pivotal role in the development and progression of gout through three core pathways: regulating microbial community structure, microbial metabolites, and intestinal barrier function. This also suggests that its associated metabolic pathways hold significant potential as therapeutic targets.

## 3 Urate-lowering substances targeting the gut microbiota

In recent years, urate-lowering agents targeting the gut microbiota have increasingly been recognized as a novel reservoir of candidate urate-lowering molecules. These substances typically possess well-defined chemical structural characteristics and exert their pharmacological effects by modulating host signaling pathways or urate transport processes, thus providing a novel entry point for the molecular design of urate-lowering drugs based on the gut microbiota. According to their modes of action, the relevant molecules can be broadly classified into three categories: microbial metabolites and functional bacterial strains, microbiota-modulating natural products, microbiota-utilizable polysaccharides, as well as Bioactive peptides.

### 3.1 Microbial metabolites and functional bacterial strains

**Fig. 2:**
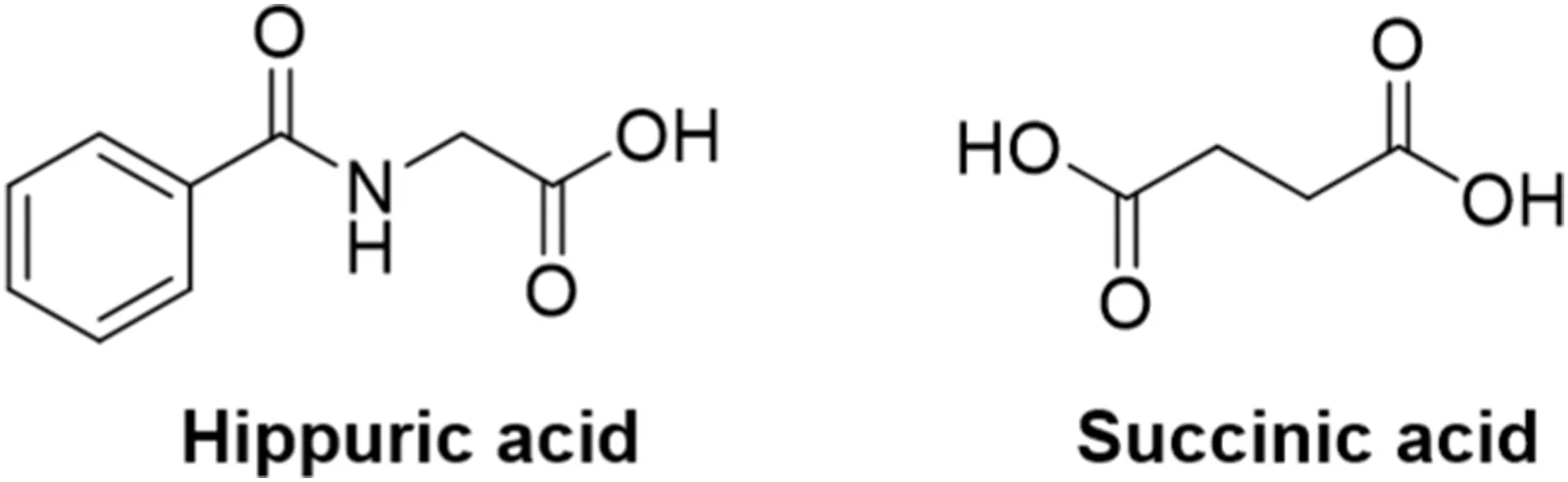
The structures of Hippuric acid and succinic acid.

In 2024, Xu et al. discovered through integrated metagenomic and metabolomic analysis that hippuric acid (Fig. 2), a metabolite derived from *Alistipes indistinctus*, is a small-molecule compound with proven urate-lowering activity. Hippuric acid is an endogenous aromatic amide compound generated through the conjugation of microbiota-derived benzoic acid with glycine in the host liver. Structurally, it contains a benzene ring linked to glycine through an amide bond, providing a simple and chemically accessible scaffold. Hippuric acid has a low molecular weight (179.17 Da), moderate water solubility, low toxicity, and high bioavailability, endowing it with favorable physicochemical properties relevant to druggability. Mechanistically, hippuric acid promotes intestinal urate excretion through activation of the PPARγ–ABCG2 axis and PDZK1-dependent membrane localization of ABCG2. In hyperuricemic mice, hippuric acid supplementation restored serum urate levels to normal ranges and increased intestinal urate excretion by approximately 60%, without significantly affecting hepatic purine metabolism or renal urate clearance. Moreover, plasma hippuric acid levels were reduced by approximately 25% in individuals with hyperuricemia compared with normouricemic controls, supporting its potential relevance in human urate homeostasis. However, further pharmacokinetic characterization and optimization are required to evaluate its translational potential. [30]. In the following year, Pan et al. reported that succinic acid (Fig. 2), a microbiota-derived short-chain dicarboxylic acid produced by *Bacteroides fragilis*, contributes to gut microbiota-mediated urate reduction. It increases hepatic phosphate levels, thereby inhibiting adenosine monophosphate deaminase 2 (AMPD2) activity and suppressing the conversion of AMP to IMP, ultimately reducing the generation of hypoxanthine, xanthine, and uric acid. In fructose-induced hyperuricemic mice, *B. fragilis* administration significantly decreased plasma uric acid levels, while fecal succinic acid abundance was increased and negatively correlated with serum urate levels. Furthermore, sodium succinate administration directly reduced serum uric acid levels and decreased the levels of downstream purine metabolites, including IMP, inosine, hypoxanthine, and xanthine, confirming its role in suppressing uric acid production at the metabolic source [9].

Besides gut microbial metabolites, functional strains themselves represent a direct strategy for microbiota-based urate intervention. Xu et al. isolated *Escherichia coli* nf-5 from healthy human intestinal samples and demonstrated its urate-degrading capability through *in vitro* metabolic assays. The strain showed efficient uric acid degradation under optimized culture conditions, with BHI medium and aerobic conditions producing the strongest activity; under these conditions, the residual uric acid concentration decreased to 0.61 mmol/L after 18 h, corresponding to a degradation rate of approximately 79.8%. Further optimization revealed that nutritional factors significantly affected its urate-degrading capacity: compared with LB medium (5.94% degradation), BHI medium enhanced urate degradation to 55.07%, whereas excessive glucose supplementation markedly inhibited this activity. These findings highlight the metabolic potential of urate-degrading functional strains as microbiota-based interventions. However, unlike microbial metabolites supported by animal or human evidence, the therapeutic efficacy, colonization stability, and safety of *E. coli* nf-5 require further validation in vivo before clinical translation [10].

*Lactobacillus* species also exhibit promising uric acid-lowering potential. *Lactobacillus rhamnosus* GG and *Lactobacillus plantarum* SQ001 can reduce serum uric acid, inhibit xanthine oxidase activity, alleviate inflammatory responses, and ameliorate gut dysbiosis, showing significant effects in hyperuricemic animal models [11,12]. A human-derived strain *Lactobacillus paracasei* M2a, isolated from the feces of healthy individuals, also possesses favorable uric acid-lowering capacity. It achieves a uric acid degradation rate of 45.53% *in vitro* and reduces serum uric acid levels by 47.88% in hyperuricemic mice [45].

Furthermore, the safety evaluation of probiotics serves as a crucial prerequisite for developing microbiome-based therapeutic strategies. Chi et al. conducted a systematic safety assessment of *Lactobacillus brevis* SLlac-19, which exhibits potential urate-lowering effects. The results demonstrated that this strain possesses excellent gastrointestinal tolerance, lacks hemolytic properties, and exhibits no acute toxicity, making it a safe candidate microorganism for future microbiota-dependent therapeutic strategies [13].

### 3.2 Microbiome-modulating natural products

Owing to their intrinsic properties of low systemic exposure and high intestinal accumulation, some natural products can specifically interact with the gut microbiota and indirectly exert urate-lowering effects by reshaping the imbalanced microbiota structure and modulating the microbial metabolic network.

**Fig. 3:**
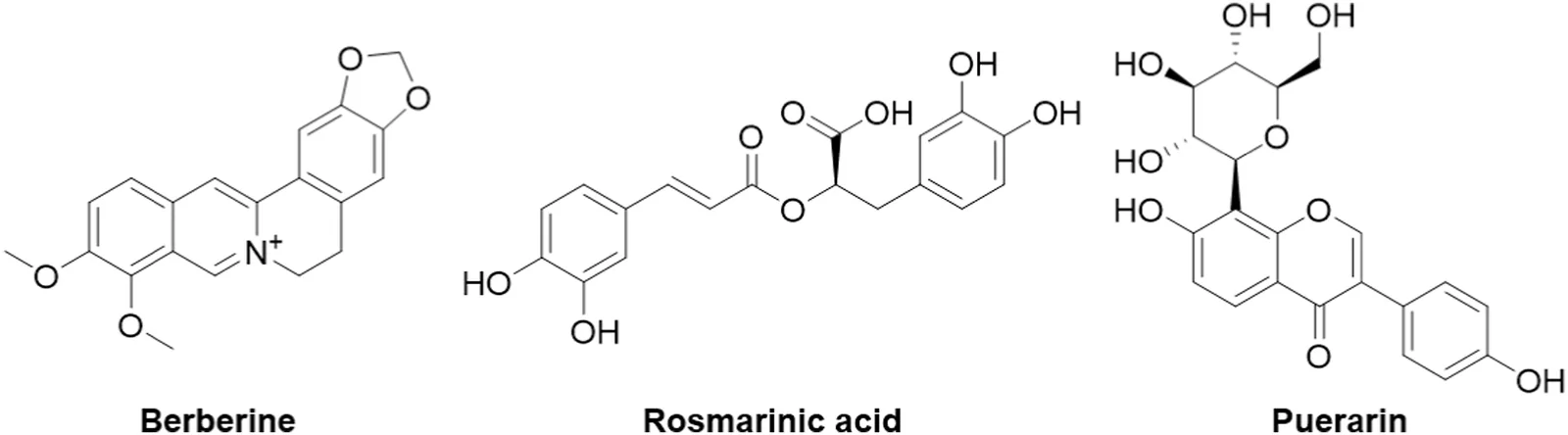
The structures of Berberine, Rosmarinic acid and Puerarin.

Berberine (BBR, Fig. 3) is a naturally occurring isoquinoline alkaloid that has been used clinically for a long time. From a medicinal chemistry perspective, BBR possesses a quaternary ammonium isoquinoline scaffold characterized by a planar aromatic ring system and a positively charged nitrogen center. These structural features limit passive membrane diffusion and contribute to its low oral bioavailability, while simultaneously promoting intestinal retention and extensive interaction with gut microbiota. Therefore, BBR represents a typical intestine-directed microbiota-modulating agent whose urate-lowering effects are mainly mediated through microbial metabolic regulation rather than direct systemic target inhibition.

In 2025, Pan et al. reported that BBR significantly reshaped gut microbial dysbiosis under hyperuricemic conditions by increasing the abundance of *Bacteroides* and decreasing *Clostridium sensu stricto*-1, thereby restoring microbial metabolic homeostasis [9]. In a high-purine diet-induced chronic hyperuricemia model in ICR mice, BBR administration produced a significant dose- and time-dependent reduction in serum uric acid levels. After 1 and 2 weeks of treatment, low-dose BBR (100 mg/kg) decreased serum urate levels by approximately 29% and 47%, respectively, whereas high-dose BBR (200 mg/kg) achieved reductions of approximately 48% and 54%, respectively. Furthermore, in a xanthine-induced acute hyperuricemia model, BBR significantly reduced serum uric acid levels with an efficacy comparable to the uricosuric agent benzbromarone. BBR also alleviated hyperuricemia-associated renal injury by reducing the kidney index and reversing elevated serum creatinine levels in a dose-dependent manner, indicating potential renoprotective effects.

Mechanistically, BBR increased the abundance of *Bacteroides* and enhanced microbial succinate production. Succinate subsequently inhibited hepatic AMPD2 activity, suppressed AMP-to-IMP conversion, and reduced downstream generation of hypoxanthine, xanthine, and uric acid. In addition, BBR inhibited microbiota-derived trimethylamine-N-oxide (TMAO) production and alleviated uric acid crystal-associated renal injury. These findings demonstrate that the low systemic exposure and high intestinal accumulation of BBR enable effective modulation of microbial metabolic networks, providing a promising scaffold for developing intestine-targeted urate-lowering agents.

Rosmarinic acid (RA, Fig. 3), a naturally occurring phenolic acid, represents another promising microbiota-targeted urate-lowering scaffold. RA exhibits limited passive membrane permeability due to its multiple phenolic hydroxyl groups and carboxyl moiety, resulting in relatively low systemic exposure and preferential interaction with intestinal microorganisms. *In vivo*, RA can be metabolized by gut microbiota into smaller phenolic metabolites, including m-coumaric acid and hydroxyphenylpropionic acid, which may contribute to its biological activity through microbial–host metabolic communication [14].

In a 10% fructose-induced hyperuricemic rat model, 28-day administration of RA significantly reduced serum uric acid levels, inhibited urate-producing enzymes including XOD and adenosine deaminase (ADA), and improved renal and intestinal dysfunction associated with hyperuricemia. Multi-omics analysis revealed that RA remodeled gut microbial composition by decreasing the *Firmicutes/Bacteroidota* ratio and increasing the abundance of *Bacteroides*, which may contribute to improved purine metabolism and urate homeostasis. Furthermore, RA regulated key urate transporters involved in urate disposition, including increasing intestinal and renal ABCG2 expression while decreasing renal URAT1 and GLUT9 expression. Molecular interaction studies further demonstrated direct binding affinities of RA toward GLUT9 (K_d_ = 6.20 μM), URAT1 (K_d_ = 667 nM), and ABCG2 (K_d_ = 264.6 nM), supporting its multitarget regulation of urate transport pathways [15].

From a medicinal chemistry perspective, RA contains a caffeic acid esterified with a hydroxyphenyllactic acid moiety, providing abundant hydroxyl groups and a carboxylic acid group. These polar functionalities reduce membrane permeability and favor intestinal retention, thereby enhancing interactions with gut microbiota. Meanwhile, the phenolic structure provides metabolic flexibility for microbial transformation, generating bioactive metabolites that may contribute to urate regulation. Therefore, phenolic acid scaffolds represented by RA provide promising templates for developing intestine-targeted urate-lowering agents; however, further optimization is required to improve metabolic stability and define the contribution of individual microbial metabolites.

Puerarin (Fig. 3), a typical C-glycosyl isoflavone, exhibits limited oral absorption due to its highly polar glycosyl structure. The C-glycosidic linkage increases chemical stability against enzymatic hydrolysis while reducing passive membrane permeability, resulting in relatively high intestinal exposure and facilitating interactions with gut microbiota. A proportion of puerarin reaches the colon in its prototype form, where it modulates microbial homeostasis and host-microbiome metabolic communication. In hyperuricemic animal models, puerarin administration has been shown to reduce serum uric acid levels by regulating gut microbiota composition, increasing SCFA-producing bacteria, improving intestinal barrier integrity, and modulating purine metabolism-related pathways [16].

Furthermore, puerarin exhibits enhanced urate-lowering activity when combined with probiotics. In potassium oxonate/adenine-induced hyperuricemic mice, co-administration of *Lactiplantibacillus plantarum* KLDS1.0328 and puerarin significantly decreased serum uric acid levels from 228.06 ± 3.57 μmol/L in the hyperuricemic model group to 38.81 ± 6.78 μmol/L, corresponding to an approximately 82.98% reduction compared with the model group [46]. This combination also suppressed serum and hepatic XOD activity, improved renal function, restored intestinal tight junction proteins (Occludin, Claudin-1, and ZO-1), inhibited NLRP3/NF-κB-mediated inflammatory responses, and reshaped gut microbial diversity [17]. Therefore, puerarin represents a promising microbiota-responsive scaffold for urate-lowering drug development, although further identification of active microbial metabolites and optimization of intestinal selectivity remain necessary.

Collectively, the therapeutic potential of microbiota-targeted natural products is closely associated with their structural features that determine intestinal exposure, microbial transformation, and host-microbiome interactions. Rather than optimizing systemic potency alone, future medicinal chemistry efforts should focus on designing intestine-directed molecules with controlled absorption, microbiota selectivity, and favorable safety profiles.

With the development of metagenomics and metabolomics, the multi-target urate-lowering mechanisms of complex natural extracts have been gradually elucidated. For example, Zhejiang Plantaginis Semen glycosides (ZPG) exhibited significant antihyperuricemic effects in a rat model of hyperuricemia induced by potassium oxonate and adenine. Administration of ZPG (100 and 400 mg/kg) significantly decreased serum UA levels, with the high-dose group reducing serum UA from 184.42 ± 22.52 μmol/L in the model group to 102.77 ± 15.94 μmol/L, accompanied by decreased serum and hepatic XOD activities. Moreover, ZPG improved renal function by reducing serum creatinine (Cr) and blood urea nitrogen (BUN) levels and alleviated renal pathological injury. Mechanistically, ZPG not only inhibited XOD activity and ameliorated lipid and purine metabolic disorders associated with hyperuricemia but also exerted microbiota-dependent effects. At the microecological level, ZPG restored gut microbial dysbiosis by regulating the Firmicutes/Bacteroidetes (F/B) ratio and enriching beneficial bacteria such as Lactobacillus, thereby contributing to improved UA metabolism and reduced inflammatory responses [18].

*Capparis spinosa* fruit (CSF) exhibited significant antihyperuricemic and renoprotective effects in potassium oxonate-induced chronic hyperuricemic C57BL/6 mice. Oral administration of CSF (200 and 400 mg/kg) markedly reduced serum and urinary UA levels, with the high-dose group achieving serum and urinary UA reduction rates of 47.90% and 53.37%, respectively. CSF treatment also decreased serum Cr, BUN, and XOD levels and alleviated renal tubular injury and inflammatory infiltration. Mechanistically, CSF inhibited IL-1β/NF-κB activation and enhanced IL-10 expression. Furthermore, 16S rRNA sequencing demonstrated that CSF modulated key bacterial taxa, including Bacteroides, Muribaculaceae, and Prevotella, suggesting its involvement in regulating the gut microbiota-immune inflammation axis during hyperuricemia progression [47]. Similarly, the ethanol extract of *Hippocampus abdominalis* improves uric acid metabolism through multiple mechanisms, including XOD inhibition, regulation of urate transporters, and restoration of gut microbiota composition [48]. *Penthorum chinense* Pursh (PCP) restores gut microbiota homeostasis under hyperuricemic conditions by modulating beneficial bacteria, including Lactobacillus and Alistipes. In addition, PCP enhances renal urate excretion and suppresses systemic inflammatory responses through inhibition of NLRP3 inflammasome activation, thereby alleviating hyperuricemia-associated renal injury [49].

*Withania coagulans* (WC), native to Pakistan, mainly promotes the production of spermidine by gut microbiota through enriching *Bifidobacterium*, thereby inhibiting the TNF signaling pathway and hepatic xanthine oxidase (XOD) activity to reduce uric acid synthesis. *Fagonia cretica* (FC) upregulates *Eubacterium xylanophilum* to promote traumatic acid generation, increases the expression of intestinal uric acid transporters ABCG2 and SLC2A9 by inhibiting the IL-17 signaling pathway, and accelerates uric acid excretion. Both can downregulate the TLR4/MyD88/NF-κB inflammatory pathway, reduce LPS and pro-inflammatory cytokine levels, and repair the intestinal tight junction barrier. They exert a significant synergistic uric acid-lowering effect when used in combination [19].

### 3.3 Microbiota-utilizable natural polysaccharides

Additionally, certain natural polysaccharides that are difficult for the host to digest but can be selectively utilized by the gut microbiota also exhibit uric acid-lowering activity.

Coix seed oligosaccharides (CSO), mainly composed of glucose units linked by *β-*1,4*-*glycosidic bonds with a degree of polymerization of 2-9, are resistant to direct hydrolysis by host digestive enzymes and can be more readily utilized by gut microbiota. In hyperuricemia models, CSO remodels gut microbiota structure and significantly increases the abundance of beneficial bacteria such as *Lactobacillus* and *Akkermansia*, as well as enriching SCFA-producing bacteria. Notably, CSO not only restores the diversity of the gut microecosystem but also significantly reverses the serum levels of lipid metabolites, including phosphatidylcholine and phosphatidylserine [20].

As a homogeneous plant-derived polysaccharide, viscum coloratum polysaccharide (VCP) significantly increases intestinal microbial α-diversity, upregulates the abundance of *Firmicutes*, and decreases the levels of *Proteobacteria* and *Actinobacteriota*. It enriches beneficial bacteria such as *Lactobacillus*, *Ruminococcus*, and *Peptostreptococcaceae*, restores the disturbed microbiota structure under hyperuricemia, and enhances intestinal uric acid degradation and excretion [21].

In addition, some marine-derived polysaccharides have also been demonstrated to reduce serum uric acid levels by selectively regulating specific functional strains. For example, fucoidan, a sulfated polysaccharide from brown algae, can selectively enrich *Bacteroides xylanisolvens* HS1, thereby activating the PPARγ signaling pathway, upregulating intestinal urate efflux transporter ABCG2 expression and enhancing intestinal urate excretion [22]. This result is consistent with the aforementioned hippuric acid mechanism, further verifying the key role of the microbiota–PPARγ–ABCG2 axis in intestinal urate clearance. It suggests that targeting the enrichment of intestinal functional strains and selectively regulating dominant strains using functional polysaccharides as regulatory carriers represents a promising novel strategy for urate-lowering therapy.

### 3.4 Bioactive peptides: a potential strategy for microbiota-mediated urate regulation

In addition to microbial metabolites, natural products, polysaccharides, and probiotics, bioactive peptides represent an emerging category of functional molecules with potential roles in microbiota-mediated urate regulation. Compared with conventional small molecules, peptides possess several unique advantages, including high target specificity, low systemic toxicity, and the ability to modulate protein–protein interactions, making them valuable resources for drug discovery and functional therapeutic development.

Food-derived bioactive peptides have demonstrated potential urate-lowering activities through direct regulation of UA metabolism, including inhibition of XOD activity and modulation of urate transport pathways.

Beyond direct regulation of urate synthetic and excretory signaling, accumulating mechanistic evidence confirms bioactive peptides remodel gut microbial homeostasis to indirectly improve hyperuricemia. Peptides serve as preferential carbon/nitrogen substrates for beneficial uricolytic bacteria in the intestinal tract, selectively enriching *Lactobacillus*, *Escherichia* urate-degrading strains and suppressing pro-inflammatory Gram-negative flora such as *Desulfovibrio*. AL-VI10 intervention significantly restored the abundance of Lactobacillaceae in aging mice feces (up 2.4-fold vs. aged model group), and enhanced intestinal microbial uric acid decomposition capacity [23]. AL-VI10, composed of hydrophobic aromatic amino acids, directly binds to endothelial membrane receptor CD44 to activate the downstream SIRT1-Nrf2 antioxidant axis, reduce intestinal ROS accumulation, maintain epithelial barrier integrity, and further boost ABCG2-mediated intestinal urate excretion.

In addition, amphibian peptide FZ1 also demonstrated microbiota-modulating capacity: when constructed into self-assembling hydrogel (RADA)4-FZ1, it elevated the ratio of anti-inflammatory M2 macrophages, reduced intestinal LPS leakage, and blocked LPS-triggered NF-κB inflammatory cascades that exacerbate vascular and renal damage under hyperuricemic conditions [50]. Structural characteristics determine the hypouricemic potential of peptides: low-molecular-weight fragments (<1000 Da) rich in tryptophan, phenylalanine and histidine show superior bioactivity.

Collectively, peptide interventions remodel gut microbial composition, optimize microbial uricolytic metabolites, protect intestinal barrier function and upregulate intestinal urate efflux transporters, forming a multi-layer synergistic regulatory network to alleviate hyperuricemia and retard gout progression. Unlike single-target chemical drugs, amphibian-derived multifunctional peptides exert simultaneous antioxidant, anti-inflammatory and microecological regulatory effects, offering complementary therapies for patients intolerant to XOD inhibitors.

However, urate-lowering peptide research remains in the early preclinical stage and faces multiple translational bottlenecks. First, systematic structural optimization of hypouricemic peptide pharmacophores is insufficient: AL-VI10 and FZ1 only have primary sequence verification, with no activity screening data for truncated fragments and amino acid substitution analogs. Second, oral peptides are easily degraded by gastrointestinal proteases, leading to poor intestinal stability and low bioavailability; most existing studies use injection administration, limiting clinical oral application. Third, the molecular interaction mechanism between peptide sequences and specific uricolytic bacteria remains unclear, as does how peptide dosage and administration cycle affect long-term microbiota homeostasis. Fourth, all validations are based on chemical-induced cell and animal hyperuricemia/aging models, lacking human clinical trial data to confirm efficacy and safety.

Future research should combine peptidomics, AI-assisted molecular design and gut microbiome multi-omics screening to optimize peptide skeletons, introduce cyclization or lipidation modification to improve intestinal stability, and develop microbiota-targeted peptide hydrogel delivery systems, to advance precision microecological therapeutic candidates for hyperuricemia and gout.

Notably, peptides can lower urate by targeting gut microbiota. Food-derived peptides act as substrates for microbial metabolism, boost beneficial bacterial growth, and regulate microbial metabolites related to uric acid homeostasis. By modulating intestinal barrier integrity and urate excretion pathways *via* microbiota, peptide interventions offer a complementary strategy to manage hyperuricemia and gout progression. However, peptide-based urate-lowering strategies are still at an early development stage, with key challenges including limited structural optimization studies, insufficient understanding of peptide absorption and intestinal stability, unclear links between peptide sequences and microbiota responses, and lack of clinical validation. Future research should focus on identifying key pharmacophores in active peptides, improving intestinal stability through rational modification, and integrating microbiome analysis with peptide design to develop precision microbiota-targeted therapies. Table 1.

**Table 1:** Summary of gut microbiota-targeted urate-lowering strategies and their mechanisms of action.

Category	Active substance	Mechanism	Antihyperuricemia activities	Ref.
Microbial metabolites	Hippuric acid (*Alistipes indistinctus)*	Activates intestinal PPARγ–ABCG2 axis and promotes ABCG2 membrane localization *via* PDZK1, enhancing intestinal urate excretion	In hyperuricemic mice, hippuric acid supplementation restored serum urate levels to the normal range and increased intestinal urate excretion by approximately 60%. Plasma hippuric acid levels were decreased by approximately 25% in hyperuricemic individuals compared with normouricemic controls.	[7]
	Succinic acid (*Bacteroides fragilis)*	Increases hepatic phosphate levels, inhibits AMPD2 activity, suppresses AMP→IMP conversion, and reduces purine-derived uric acid synthesis	*B. fragilis* supplementation decreased serum UA in fructose-induced hyperuricemic mice; sodium succinate reduced UA and downstream purine metabolites	[8]
Functional Bacterial Strains	*Escherichia coli* nf-5	Directly degrades uric acid through microbial uricolytic activity; degradation capacity regulated by oxygen and nutrient availability	Under optimized aerobic BHI culture conditions, residual uric acid decreased to 0.61 mmol/L after 18 h, corresponding to approximately 79.8% degradation *in vitro*.	[9]
	*Lactobacillus rhamnosus* GG/*Lactobacillus plantarum* SQ001	Modulates gut microbiota, inhibits XO activity, and alleviates inflammation	Reduced serum UA and improved dysbiosis in hyperuricemic animal models	[10,11]
	*Lactobacillus paracasei* M2a	Degrades uric acid and regulates intestinal microbial homeostasis	Exhibited 45.53% uric acid degradation *in vitro* and reduced serum uric acid levels by 47.88% in hyperuricemic mice.	[12]
Natural products	Berberine	Reshapes gut microbiota, increases *Bacteroides* abundance, elevates succinate production, suppresses TMAO generation, and inhibits uric acid synthesis	In high-purine diet-induced chronic hyperuricemic ICR mice, BBR (100 and 200 mg/kg) reduced serum uric acid levels in a dose- and time-dependent manner: low-dose BBR decreased SUA by 29% and 47% after 1 and 2 weeks, whereas high-dose BBR achieved 48% and 54% reductions, respectively.	[8]
	Rosmarinic acid	Remodels gut microbiota by decreasing F/B ratio and increasing Bacteroides abundance; regulates purine-related metabolites; modulates urate transporters including ABCG2, URAT1, and GLUT9	In 10% fructose-induced hyperuricemic rats, 28-day RA administration significantly reduced serum UA levels, decreased XOD and ADA activities, improved renal and intestinal dysfunction, and restored gut microbiota imbalance. RA showed binding affinity toward GLUT9 (*K*_d_ = 6.20 μM), URAT1 (*K*_d_ = 667 nM), and ABCG2 (*K*_d_ = 264.6 nM)	[13,14]
	Puerarin	Modulates gut microbiota, enriches SCFA-producing bacteria, improves intestinal barrier integrity, and regulates purine metabolism	In potassium oxonate/adenine-induced hyperuricemic mice, combined puerarin and *L. plantarum* KLDS1.0328 reduced serum UA from 228.06 ± 3.57 μmol/L (HUA model) to 38.81 ± 6.78 μmol/L, achieving approximately 82.98% reduction. The combination also inhibited serum/hepatic XOD activity and improved renal injury markers.	[15,16]
	ZPG	Inhibits XOD, regulates purine/lipid metabolism, restores microbiota balance, and enriches beneficial bacteria	400 mg/kg group reduced serum UA from 184.42 μmol/L in the model group to 102.77 μmol/L	[17]
	CSF	Modulates gut microbiota, inhibits IL-1β/NF-κB inflammation, and enhances IL-10 signaling	400 mg/kg group achieveda serum UA reduction rate of 47.90% and a urinary UA reduction rate of 53.37%.	[18]
Polysaccharides	CSO	Fermented by gut microbiota, enriches *Lactobacillus* and *Akkermansia*, increases SCFA production	Reduced serum UA and reversed hyperuricemia-associated microbiota imbalance in animal models	[19]
	VCP	Remodels microbiota composition, enriches beneficial bacteria, and promotes intestinal urate metabolism	Improved disturbed microbiota structure and enhanced intestinal urate elimination	[20]
	Fucoidan	Enriches *Bacteroides xylanisolvens* HS1, activates PPARγ signaling, and increases intestinal ABCG2 expression	Reduced serum UA through microbiota-dependent regulation of intestinal urate transport	[21]
Bioactive peptides	AL-VI10 peptide	Remodels gut microbiota, increases Lactobacillaceae abundance, activates SIRT1–Nrf2 pathway, and enhances ABCG2-mediated urate excretion	Increased Lactobacillaceae abundance (∼2.4-fold) and enhanced intestinal microbial urate degradation capacity	[22]
	(RADA)4-FZ1 hydrogel peptide	Regulates microbiota, reduces LPS leakage, increases M2 macrophages, and inhibits NF-κB inflammation	Alleviated hyperuricemia-associated inflammatory injury in experimental models	[23]

In summary, existing studies indicate that urate-lowering bioactive substances mediated by the gut microbiota mainly come from four main structural categories: gut microbiota metabolites and functional strains, natural products, polysaccharides, and bioactive peptides. These molecules typically show low systemic exposure, high local intestinal concentration, and notable targeted effects. Their urate-lowering actions mainly result from reshaping gut microbiota structure and regulating microbial metabolic profiles, which in turn influence the expression of host urate transporters and inflammation-related signaling pathways. ABCG2 and its upstream regulator PPARγ serve as key nodes through which most small molecules ultimately exert their uric acid excretion-promoting effects. Whether they are microbial metabolites such as hippuric acid and succinic acid, or natural small molecules including RA and BBR, their pharmacological effects depend to varying degrees on the modulation of PPARγ activity and the transcriptional upregulation of ABCG2. This indicates that the "microbial metabolite/natural product–host transporter target" axis constitutes an important effector pathway for microbiota-targeted interventions in hyperuricemia and also provides a clear target basis for subsequent drug design.

From a medicinal chemistry perspective, these molecules share several common structural features. First, these bioactive substances generally exhibit favorable intestinal compatibility and diverse chemical features, including carboxyl, hydroxyl, amide, and peptide bond-containing structures. Second, these molecules generally possess small to moderate molecular weights and relatively simple skeletons, such as aromatic acid, polyphenol, or isoquinoline moieties, showing favorable drug-likeness and structural modifiability. In addition, some natural products are inherently characterized by low intestinal absorption, allowing high local enrichment in the intestine and thereby achieving a microbiota-targeted local mode of action while reducing potential systemic side effects.

Accordingly, urate-lowering active substances targeting the gut microbiota can be regarded as a promising and underexplored resource for novel anti-hyperuricemic drug discovery. Their structural diversity and unique mode of action provide promising lead scaffolds and design rationales for next-generation urate-lowering agents. Future research should focus on the structural elucidation and target identification of gut microbiota-derived metabolites, establish drug design strategies centered on microbiota-associated signaling axes, and achieve synergistic improvements in the safety and efficacy of active substances through structural optimization. This will further drive the paradigm shift of urate-lowering drug development from traditional enzyme inhibition toward a new model of microbiota-mediated systemic regulation.

## 4 Summary and Outlook

In summary, the pathogenesis of hyperuricemia has shifted from viewing it as a simple disorder of purine metabolism to recognizing it as a systemic pathological process involving the gut, kidneys, and systemic metabolic networks. As a core regulatory hub, the gut microbiota plays a multidimensional role in controlling UA production, degradation, excretion, and inflammatory responses through a regulatory network of “microecology-metabolic pathways-host targets”. Existing studies have demonstrated that microbiota-targeted urate-lowering strategies possess unique advantages, including potential synergistic urate-lowering and anti-inflammatory effects, enhanced intestinal targeting, and reduced systemic exposure. This multi-level regulatory mode provides a complementary approach to conventional XOD inhibitors and urate transporter modulators and represents an emerging direction for the development of novel urate-lowering therapies.

Despite rapid advances in microbiome research, small molecules targeting gut microbiota for urate reduction are still at early development stages from a medicinal chemistry perspective. The targets and structure-activity relationships of bioactive molecules remain understudied, and the chemical basis of microbiota-dependent efficacy requires further investigation. Additionally, the metabolic pathways, pharmacokinetic properties, and in vivo exposure profiles of microbial metabolites need systematic characterization.

Notably, substantial interindividual variation in gut microbiota composition, shaped by genetics, diet, lifestyle, geography and medication history, can lead to heterogeneous therapeutic responses among patients. Thus, identifying reliable microbiota signatures and validated biomarkers for patient stratification, treatment monitoring and efficacy prediction is a key challenge for clinical translation.

Regulatory challenges for live biotherapeutics (including probiotic strains and engineered microorganisms) also require careful consideration. Issues of strain identification, genetic stability, manufacturing consistency, quality control, and long-term gut ecological effects demand systematic evaluation to guarantee safety and reproducibility.

Moreover, standardized, clinically relevant models are urgently needed to assess microbiota-dependent efficacy, since conventional animal models cannot fully replicate the complexity and variability of the human gut microbiome. Humanized microbiota models, intestinal organoid systems, and integrated microbiome-host evaluation platforms offer valuable tools for mechanism validation and clinical translation.

In the future, research and development of urate-lowering drugs based on the gut microbiota can proceed in the following specific directions:

i. Taking microbial metabolites such as hippuric acid and succinic acid as the core lead scaffolds, conduct systematic research on structure-activity relationships, and focus on modifying key functional groups including amide bonds and carboxyl groups to enhance their PPARγ activation activity and intestinal stability. On this basis, safer molecular optimization may be achieved by using clinical PPARγ agonists such as rosiglitazone as references, reducing excessive systemic agonism while improving intestinal selectivity. Meanwhile, flavonoid small molecules such as luteolin and naringenin exert uric acid-lowering and anti-inflammatory effects by inhibiting CDK5-dependent phosphorylation of PPARγ at Ser273, providing natural lead structure references for PPARγ pathway-targeted drugs [51].
ii. With the emergence of intestine-selective GLUT9 inhibitors such as SG4, GLUT9 has attracted increasing attention as an important urate transporter in intestinal epithelial cells [52]. As another important high-affinity uric acid transporter in small intestinal epithelium, inhibition of GLUT9 can significantly enhance the intestinal excretory pathway, and this strategy can complement the PPARγ–ABCG2 excretion pathway driven by microbial metabolites. Future efforts should focus on designing molecules with low systemic exposure, intestinal enrichment, and microbiota compatibility to achieve effective and safe urate regulation.
iii. Microbiota-responsive prodrugs represent another promising strategy. By utilizing gut microbiota-specific enzymes, such as β-glucosidase and esterase, these prodrugs may achieve site-specific release of active molecules within the intestinal tract, thereby improving targeting efficiency and reducing systemic exposure.
iv. Developing dual-functional molecules that simultaneously modulate microbiota activity and regulate urate transport may provide synergistic therapeutic effects. For example, conjugating structural motifs derived from SCFAs with ABCG2 modulators may achieve a combined effect of “microbiota regulation + direct promotion of urate excretion”.
v. Gut microbiota characteristics may serve as potential ecological biomarkers for predicting hyperuricemia and gout susceptibility or therapeutic response. In the future, integrating high-throughput sequencing, metabolomics, and machine learning approaches to establish microbiome-based predictive models, together with probiotic formulations and microbiota modulators, may enable personalized interventions for uric acid metabolism [53].

Meanwhile, further efforts should focus on improving functional bacterial strains through genetic engineering approaches, such as enhancing urate degradation capacity in *Escherichia coli* nf-5, and developing intestinal-targeted delivery technologies. These advances may accelerate the transition from basic research to clinical application and promote the development of microbiota-based urate-lowering therapies.

## CRediT authorship contribution statement

**Yuxin Jiang:** Writing – original draft, Validation, Methodology, Investigation, Formal analysis, Conceptualization. **Xiaoyu Shi:** Writing – review & editing, Conceptualization. **Qian Yang:** Writing – review & editing, Validation. **Jianxin Pang:** Writing – review & editing, Conceptualization. **Peng Zhan:** Writing – review & editing, Supervision, Project administration, Funding acquisition, Conceptualization. **Shujing Xu:** Writing – review & editing, Supervision, Conceptualization. **Ting Wu:** Writing – review & editing, Supervision, Conceptualization.

## Data availability

Not applicable.

## Ethics approval

Not applicable.

## Declaration of generative AI in scientific writing

No generative AI tools have been used throughout the entire writing process of this manuscript.

## Funding information

We gratefully acknowledge financial support from the National Natural Science Foundation of China (U25A20175; 82473769; T2321004; 82373921). This research was also supported by the State Key Laboratory of Natural and Biomimetic Drugs (KF2025020), State Key Laboratory of Druggability Evaluation and Systematic Translational Medicine (Tianjin Institute of Pharmaceutical Research) (712025001), and the Independent Project of Shandong Basic Research Special Zone (Pharmacy) (Grant No. TQ052025001).

## Declaration of competing interest

The authors declare that they have no known competing financial interests or personal relationships that could have appeared to influence the work reported in this paper.
